# The information requirements and self-perceptions of Turkish women undergoing hysterectomy

**DOI:** 10.12669/pjms.321.7962

**Published:** 2016

**Authors:** Emine Gercek, Nursel Alp Dal, Hande Dag, Seyran Senveli

**Affiliations:** 1 Emine Gercek, PhD, RN, Assistant Professor, Nursing Department, Adnan Menderes University, Soke School of Health, Soke, Aydin, Turkey; 2 Nursel Alp Dal, MsC, Lecturer, Nursing Department, Tunceli University, Tunceli School of Health, Tunceli, Turkey; 3 Hande Dag, PhD, Research Assistant, Obstetrics and Gynecologic Nursing Department, Ege University, Faculty of Nursing, Bornova, Izmir, Turkey; 4 Seyran Senveli, Lecturer, Nursing Department, Canakkale Onsekiz Mart University, School of Health, Canakkale, Turkey

**Keywords:** Pre and Post-Hysterectomy, Discharge, Information Needs, Psychological Status

## Abstract

**Objectives:**

To investigate the affects, information requirements and self-perceptions of Turkish women undergoing hysterectomy.

**Methods:**

A descriptive cross-sectional study was conducted on 37 Turkish women undergoing hysterectomy and followed in a gynecology unit of a state hospital in Canakkale, Turkey, between February and August 2012. Data were collected before discharge with a questionnaire composed of 32 questions. Percentage distributions and Chi-square test were used in the evaluation of the data.

**Results:**

There was a significant relationship between fear of anesthesia and number of pregnancies (p=0.007) and between death during surgery and number of pregnancies in the preoperative period (p=0.027). The relationship between knowing type of surgery and knowing when sutures would be removed was also significant in post-operative period (p=0.045). In addition, there was a significant relationship between women’s living only with their husbands and worrying about not having children anymore (p=0.032).

**Conclusion:**

The women’s information needs were high and women’s self-perceptions had been affected negatively after hysterectomy. It is recommended that nurses, primarily health professionals should have adequate knowledge on comprehensive care and psychosocial support after hysterectomy.

## INTRODUCTION

Hysterectomy is the second most commonly performed gynecological surgical procedures after cesarean section.^1^ National Center for Health Statistics in the United States had reported that hysterectomy was performed in about 600,000 women between 2000-2004. This operation was performed more frequently than other age groups to women who were in the 40-44 age groups.^1^,^2^ It is estimated that hysterectomy had been performed on more than 200,000 women in Turkey.^3^

Hysterectomy is a major surgical procedure that brings in significant physiological and psychological complications.^1^ Although it is well known that hysterectomy may affect self-perceptions and self-esteem of women, it is emphasized that the sexual function of women who have to maintain their female identity, values and sexuality after hysterectomy are neglected by health professionals.^4^,^5^

It is stated that the women who have experienced hysterectomy have fear of losing the sexual function, reproductive capability and femininity role.^6^,^7^ Apart from these worries, these women have fears related to harmful effects of menopause, lose their physical strength and have a poor relationship with their spouses.^8^ The main cause of these worries and fears is that women’s information needs before and after hysterectomy are not met effectively and that appropriate interventions (effective communication, providing social support, behavioral approaches etc.) are not performed.^5^ In a study, it was found that the most common causes of fear of the women before hysterectomy were mistakes related to which organs will be removed, fear of not waking up after hysterectomy, not receiving adequate care and attention from health professionals.^7^

At present, women undergoing hysterectomy stay for a shorter period of time in hospital and experience the healing process in their homes. In several studies, it has been showed that women want information and advices on type of surgery, difficulties that may be encountered after surgery, the possible complications of the hysterectomy, problems in course of healing process and care. Therefore, providing training about needs before and after hysterectomy and evaluating femininity perceptions of women undergoing hysterectomy is of great importance for comprehensive nursing care. The aim of the study was to examine information requirements pre and post hysterectomy and self-perceptions after hysterectomy of the Turkish women.

## METHODS

This was a descriptive and cross-sectional study. It was conducted at the gynecology clinic of a state hospital in Canakkale, Turkey, between February and August 2012. The inclusion criteria of this study included; undergoing total abdominal hysterectomy and bilateral salpingo oophorectomy (TAH+BSO), total abdominal hysterectomy (TAH) and vaginal abdominal hysterectomy (VAH) as type of surgery, having no perioperative and early postoperative complications, before or at the time having no diagnosed psychiatric disorders, having no seeing or hearing problems, being open to communication and cooperation, voluntarily accepting to participate in the study and giving written informed consent. Of 52 women, 9 declined to participate in the study and 6 did not meet the inclusion criteria. The sample of the study thus consisted of 37 women.

### Data collection

Data were collected with a questionnaire prepared by the researchers in the light of published literature. The questionnaire was composed of 9 questions regarding the descriptive features of women (age, education, marital status, income, employment status, people with whom women live), obstetrics (the number of pregnancies) and gynecological history (type of surgery, menopausal status) and 23 questions regarding pre-operative and post-operative needs and psychological status. The questionnaire’s content validity was evaluated by five teaching members who are expert in nursing and medical sciences. Each item in the questionnaire was scored between one and three points by experts. The grading was performed to measure the degree of each question in the questionnaire as “1 = item is required,” “2 = item is useful but not enough substance,” “3 = item is not required. The content validity was considered acceptable (Content validity index=0.86; α=0.05). The questionnaire was applied to test readability and comprehensibility to 15 women who were not included in the present study by the researchers. The necessary adjustments on the questionnaire were made in accordance with recommendations of the women. The data were obtained with face-to-face interviews in the post-operative period. The participants were given approximately 20 minutes to answer the questions.

### Statistical Analysis

Statistical analyses were performed by using the Statistical Package Program for Social Sciences, version 18.0 (SPSS, Inc., Chicago, IL). Descriptive statistics including frequency and percentage were used to describe demographic, obstetric and gynecologic characteristics of the women. Also, frequency distributions for pre and post-operative needs and for descriptive features of psychological status were determined. Chi-square test was used to evaluate factors affecting psychological status and pre and postoperative needs of women undergoing hysterectomy.

### Ethical approval

Written permission from ethical committee and approval from the institution where the study was performed were obtained prior to the study. The women were informed on the objective of the study and their informed consent was obtained.

## RESULTS

The participants’ descriptive, obstetrics and gynecologic characteristics are shown in Table-I. The mean age of the women participating in this study was 50.62±1.16 years. Of 37 women included in the study, 56.8% were primary education graduates, 78.4% had an income equal to their expenses, 78.4% were housewives, 67.6% were living with their spouse and children and 51,4% became pregnant twice. Of all the women, 59.5% had TAH and 51.4% had surgical menopause.

**Table-I:** Characteristics of women undergoing hysterectomy (N=37).

*Variables*	N	%
*Age groups*		
40-49 years	23	62.2
50 years and over	14	37.8
*Education*		
Literate	5	13.5
Primary education	21	56.8
High school or a higher level of education	11	29.7
*Marital status*		
Married	29	78.4
Single	8	21.6
*Income*		
Income lower than expenses	8	21.6
Income equal to expenses	29	78.4
*Family members with whom women stayed*		
Spouse	12	32.4
Spouse and children	25	67.6
*Employment status*		
Employed	8	21.6
Unemployed	29	78.4
*Number of pregnancies*		
1	7	18.9
2	19	51.4
3 and over	11	29.7
*Type of surgery*		
VAH	6	16.2
TAH	22	59.5
TAH+ BSO	9	24.3
*Menopausal status*		
Menopause before surgery	13	35.1
Menopause after surgery	19	51.4
Not having menopause	5	13.5

Total	37	100

The information needs in pre and post-operative period of the women are shown in Table-II. Eighty-one point one percent of the women were found to know about the type of surgery and which organs to be removed before surgery, 86.5% knew about locations and functions of the organs to be removed and 62.2% were found to know about treatment to be carried out after surgery and its side-effects. Of all the women, 75.7% noted that they knew lifting heavy objects would damage their surgical wound, 67.6% noted that they knew when their sutures would be removed and 67,6% did not know when they could start to have a sexual relationship.

**Table-II:** Information requirements of womenbefore and after hysterectomy

	N	%
Before hysterectomy		
I had the fear of anesthesia before surgery.	16	43.2
I had the fear of dying due to surgery.	16	43.2
I know what type of surgery was performed and which of my organs were removed.	30	81.1
I know the location and functions of the organs removed.	32	86.5
I know about the treatment to be implemented after surgery and its effects and side-effects.	23	62.2
After hysterectomy		
I know when I have to come to hospital for the follow-up.	21	56.8
I know when the sutures will be removed.	25	67.6
I know lifting heavy objects causes damage to the surgical wound.	28	75.7
I know what I should care about when I go back home (increased temperature, redness around the surgical wound, leakage, hernia, pain).	19	51.4
I know when I can have a bath.	19	51.4
I know when I can have a sexual relationship.	12	32.4

* More option than one had been marked.

Sixty-seven point six percent of the women felt tired and 64.9% had difficulty in falling asleep. Sixty-four point nine percent of the women said they felt like a man, 54.1% were worried that they would not have children any longer and 83.8% of the women thought that they would be able to fulfill their religious duties more easily (Table-III).

**Table-III:** The self-perceptions of the women after hysterectomy.

	N	%
I feel tired.	25	67.6
I have difficulty in falling asleep.	24	64.9
I feel unhappy after surgery.	17	45.9
I feel as I were a man.	24	64.9
I fear that my disease will deteriorate and turn into other diseases.	18	48.6
I am upset about not having a child any more.	17	45.9
I am happy because I will be able to fulfill my religious duties more comfortably.	31	83.8
I feel as if there were a hole in my body.	10	27.0
I think, as a woman I have lost an important part of my body.	12	32.4
I feel as if I were not a whole person.	8	21.6

* More option than one had been marked.

Concerning factors affecting information needs of the women before hysterectomy, there was a significant relation between fear of anesthesia and number of pregnancies (p=0.007) and between possibility of death during surgery and number of pregnancies (p=0.027). In addition, there was a significant relation between family members with whom the women lived and type of surgery (p=0.025) and knowing about functions of the organs to be removed (p=0.030). The relation between type of surgery and knowing about the treatment method performed and its side-effects was also significant (p=0.029; Table-IV).

**Table-IV:** Factors affecting preoperative information requirements of the women undergoing hysterectomy.

Variables	Fear of anesthesia				Fear of death during surgery				Information of type of surgery and the organs removed				Information of functions of the organs removed				Information of treatment to be given after surgery and its side-effects			

	Yes		No		Yes		No		Yes		No		Yes		No		Yes		No	

	N	%	N	%	N	%	N	%	N	%	N	%	N	%	N	%	N	%	N	%
Marital status																				
Married	11	34.5	18	65.5	10	34.5	19	65.5	24	82.8	5	17.2	26	89.7	3	10.3	19	65.5	10	34.5
Single	5	75.0	3	25.0	6	75.0	2	25.0	6	64.9	2	13.5	6	75.0	2	25.0	4	50.0	4	50.0
*X^2^*	1.520^¶^				4.194^¶^				0.246^¶^				1.152^¶^				0.642^¶^			
P	0.254				0.054*				0.631				0.292				0.445			
Family members with whom the women stayed																				
Eş	6	50.0	6	50.0	7	58.3	5	41.7	7	58.3	5	41.7	8	66.7	4	33.3	7	58.3	5	41.7
Eşveçocuklar	10	40.0	15	60.0	9	36.0	16	64.0	23	92.0	2	8.0	24	96.0	1	4.0	16	64.0	9	36.0
*X^2^*	0.330^¶^				1.648^¶^				5.991^¶^				5.969^¶^				0.111^¶^			
P	0.565				0.199				0.025*				0.030*				0.739			
Number of Pregnancies																				
1 gebelik	0	0.0	7	100.0	0	0.0	7 8	100.0	5	71.4	2	28.6	5	71.4	2	28.6	3	42.9	4	57.1
2 gebelik	8	42.1	11	57.9	11	57.9	8	42.1	14	73.7	5	26.3	18	94.7	1	5.3	15	78.9	4	21.1
3 veüzerigebelik	8	72.7	3	27.3	5	45.5	6	54.5	11	100.0	0	0.0	9	81.8	2	18.2	5	45.5	6	54.5
*X^2^*	9.336^¶^				7.277^¶^				3.879				2.918				4.700			
P	0.007*				0.027*				0.115				0.240				0.108			
Type of Surgery																				
VAH	2	33.3	4	66.7	2	33.3	4	66.7	5	83.3	1	16.7	6	100.0	0	0.0	6	100.0	0	0.0
TAH	9	40.9	13	59.1	10	45.5	12	54.5	17	77.3	5	22.7	19	86.4	3	13.6	10	45.5	12	54.5
TAH+ BSO	5	55.6	4	44.4	4	44.4	5	55.6	8	88.9	1	11.1	7	77.8	2	22.2	7	77.8	2	22.2
*X^2^*	0.889^¶^				0.374^¶^				0.564^¶^				1.273^¶^				6.889^¶^			
P	0.723				0.903				0.847				0.658				0.029*			
Menopausal Status Ameliyattanöncegiren Ameliyatilebirliktegiren Yalnız uterus alındığıiçinhalagirmeyen	9 5 2	69.2 26.3 40.0	4 14 3	30.8 73.7 60.0	7 6 3	53.8 31.6 60.0	6 13 2	46.2 68.4 40.0	10 15 5	76.9 78.9 100.0	3 4 0	23.1 21.1 0.0	10 17 5	76.9 89.5 100.0	3 2 0	23.1 10.5 0.0	10 10 3	76.9 52.6 60.0	3 9 2	23.1 47.4 40.0
Menopausal Status Ameliyattanöncegiren																				
Ameliyatilebirliktegiren	9	69.2	4	30.8	7	53.8	6	46.2	10	76.9	3	23.1	10	76.9	3	23.1	10	76.9	3	23.1
Yalnız uterus	5	26.3	14	73.7	6	31.6	13	68.4	15	78.9	4	21.1	17	89.5	2	10.5	10	52.6	9	47.4
alındığıiçinhalagirmeyen	2	40.0	3	60.0	3	60.0	2	40.0	5	100.0	0	0.0	5	100.0	0	0.0	3	60.0	2	40.0
*X^2^*	5.711^¶^				2.265^¶^				1.023^¶^				1.485				1.992^¶^			
P	0.052				0.336				0.719				0.414				0.408			

* *P*< 0.05; VAH: Vaginal Abdominal Hysterectomy, TAH: Total Abdominal Hysterectomy,

TAH+BSO: Total Abdominal Hysterectomy and Bilateral Salpingo Oophorectomy,

¶ Fisher’s Chi-square test was used. Those highlighted in Red should be changed into English

As regards factors affecting post-operative information needs of the women, there was a significant relation between education levels and which doctor (i.e. an oncologist, radiologist or an obstetrician etc.) they had to see after surgery (p=0.013) and knowing when to have a bath (p=0.014). The relation between knowing about type of surgery and knowing when their sutures to be removed (p=0.045).

Concerning psychological status of the women undergoing hysterectomy, there was a significant relation between being married and women’s feeling as if they were a man (p=0.032). The relation between women’s living only with their spouses and thinking that they would not have a child any more was also significant (p=0.032).

## DISCUSSION

The most important finding of the present study was that information needs before hysterectomy were fulfilled in most of the women, but that information needs after hysterectomy were not fulfilled in half of the women. In addition, the present study also revealed that hysterectomy had a negative impact on the women’s psychology. Likewise, in the studies reported so far, women undergoing hysterectomy noted that they lost some part of their sexual life, they felt as if there were a great hole in their body and as if they were not a whole person and they were worried about their sexual life and loss of their reproductive ability.^5^,^9^-^11^ Besides, many studies have revealed negative emotional outcomes following hysterectomy.^11^,^12^ In the present study, most of the women felt tired and had difficulty in falling asleep, indicative of psychological effects of hysterectomy. Similarly, in a study by Carlson, Miller, and Fowler, 91% of 271 women noted that they had fatigue after abdominal hysterectomy.^13^ Kim and Lee showed that sleep disturbance and fatigue persisted in the women undergoing vaginal hysterectomy 6 weeks after surgery. Postoperative sleep disturbance can contribute to fatigue^14^, which has been reported to be a major symptom following hysterectomy and cause difficulty in daily activities.^15^

Wade in their study found that women who would undergo hysterectomy wanted to have information about anatomy and physiology of the uterus, hospital and surgical procedures, anesthesia, physical, emotional and emotional changes, symptoms after hysterectomy and hormone replacement therapy.^16^ Consistent with the results of the present study, as cited by Karadağ and Sabuncu, Black and Lewis found that women undergoing hysterectomy needed information about pain, wound care, restriction of activities, nutrition, using medications at home, ways of prevention of postoperative complications, personal hygiene and regular physicals.^17^ The present study also revealed that physical effects of hysterectomy were common and therefore the women needed information about physical care. It is obvious that symptoms of patients should be routinely evaluated and appropriate education should be offered on discharge to achieve postoperative care management. In the study by Güler and Taşkın is stated that the women could have less problems if we find a solution to problems encountered during training related to the healing process prior to discharge.^18^

The current study showed that family members with whom the women stayed, number of pregnancies and type of surgery played an important role in the women’s preoperative information needs. Besides, the women’s postoperative information needs were influenced by their education level and type of surgery and psychological status was influenced by marital status and family members with whom the women stayed. Likewise, in a study by Harlow and Barbieri, women with only a high school education turned out to be about four times more likely to have hysterectomy and need education about hysterectomy than those with a college degree or a higher level of education.^19^ In contrast to the findings from the present study, Ozdemir and Pasinlioglu have reported no significant differences between type of surgery and preoperative and postoperative opinions about hysterectomy.^10^ In a study by Wong and Arumugam no relation was found between psychological impacts in the post-hysterectomy period and marital status and employment status.^15^

In the present study, not having sufficient information before hysterectomy was found to affect preoperative and postoperative information needs. Since uterus is an important organ symbolically throughout lives of most women, hysterectomy can be traumatic. Although there are apparently few changes after hysterectomy, perceived self, self-confidence and body image of women can be affected radically. Therefore, women undergoing hysterectomy experience physical and psychological stress and fear of worse sexual life and cessation of sexual life. Meta-analyses has revealed that interventions such as education for information and skills and psychosocial support contribute to positive postoperative outcomes.^20^,^21^

Based on the findings of the present study, it can be suggested that marital status and social support from family members with whom the women stayed influenced psychology of the women. Support especially from spouses and children can be effective in coping with psychological problems caused by hysterectomy. It has also been reported that well-organized postoperative education programs offered to women having hysterectomy will decrease postoperative anxiety and pain, enhance self-care behavior and eliminate negative effects on perceived sexuality.^22^ Nurses working in gynecology and obstetrics clinics play an important role in offering counseling for the women undergoing hysterectomy. Preoperative counseling and interventions to be offered by nurses will help women to cope with negative outcomes of surgery. Therefore, training about the procedures to be performed before and after surgery, complications of hysterectomy, healing process, and care of the women undergoing hysterectomy is of great importance for appropriate nursing care.

### Limitations

The study sample was the small hence the research findings cannot be generalized.

## CONCLUSION

Information needs of women after hysterectomy were not fulfilled completely and hysterectomy had a negative effect on women’s self-perceptions. Type of surgery also had an influence on pre and post-operative information needs and social support influenced self-perceptions and pre-operative information needs in the women undergoing hysterectomy. Therefore, it is of great importance that comprehensive and individual training should be given by health professionals to increase women’s quality of life after discharge.

It is recommended that primarily nurses working in the gynecology clinics should be trained about women’s life after hysterectomy. The health care providers should emphasize the importance of social support to women and families before and after hysterectomy. In addition, it is suggested that the longer-term studies related to women’s self-perceptions and information requirements of those undergoing hysterectomy are performed.
